# Comparing modified Lee and White method against 20-minute whole blood clotting test as bedside coagulation screening test in snake envenomation victims

**DOI:** 10.1590/1678-9199-JVATITD-2022-0088

**Published:** 2023-06-19

**Authors:** Appu Suseel, Siju V. Abraham, Sarah Paul, Maglin Monica Lisa Tomy, Aboobacker Mohamed Rafi

**Affiliations:** 1 Department of Emergency Medicine, Jubilee Mission Medical College and Research Institute, Thrissur, Kerala, India.; 2 Department of Immunohematology and Blood Transfusion, Jubilee Mission Medical College and Research Institute, Thrissur, Kerala, India.

**Keywords:** 20-minute whole blood clotting test, Modified Lee and White method, Venom-induced consumption coagulopathy, Conventional coagulation tests, Prothrombin time

## Abstract

**Background:**

Twenty-minute whole blood clotting test (20WBCT) and Modified Lee and White (MLW) method are the most routinely employed bedside tests for detecting coagulopathic snake envenomation. Our study compared the diagnostic utility of MLW and 20WBCT for snakebite victims at a tertiary care hospital in Central Kerala, South India.

**Methods:**

This single-center study recruited 267 patients admitted with snake bites. 20WBCT and MLW were performed simultaneously at admission along with the measurement of Prothrombin Time (PT). The diagnostic utility of 20WBCT and MLW was determined by comparing the sensitivity (Sn), specificity (Sp), positive and negative predictive values, likelihood ratios, and accuracy at admission with an INR value > 1.4.

**Results:**

Out of 267 patients, 20 (7.5%) patients had VICC. Amongst those who had venom-induced consumption coagulopathy (VICC), MLW was prolonged for 17 patients, (Sn 85% 95% confidence interval [CI]: 61.1-96.0) whereas 20WBCT was abnormal for 11 patients (Sn 55%, 95% CI: 32.04-76.17). MLW and 20WBCT were falsely positive for the same patient (Sp 99.6%, 95% CI: 97.4-99.9%).

**Conclusion:**

MLW is more sensitive than 20WBCT to detect coagulopathy at the bedside amongst snakebite victims. However, further studies are necessary for standardizing bedside coagulation tests in snakebite cases.

## Background

Snakebite is a neglected disease of the poor population in India [ 1]. India is the largest contributor to the global burden of snakebite in terms of mortality with more than 50,000 deaths due to snakebite every year [ 2, 3]. Mortality due to snakebite in India is mostly due to snake species that cause venom-induced consumption coagulopathy (VICC), the most common species involved being the Russell's Viper ( *Daboia russelii*) [ 3]. In the absence of a reliable bedside venom detection kit, clinicians rely on the patient's symptomatology, clinical signs, and laboratory detection of coagulopathy to determine the presence of envenomation and the need for snake antivenom (ASV) [ 4]. 

Various methods are available for bedside detection of coagulopathy, with the most commonly used being the twenty-minute whole blood clotting test (20WBCT), modified Lee and White (MLW), and venous clotting time (VCT) [ 4]. These tests, which are modifications of Lee and White's original description of clotting time (LWCT), offer the potential advantages of being inexpensive, requiring limited resources, and being easily reproducible [ 4, 5]. The limited availability of more standardizable conventional coagulation tests (CCT) such as prothrombin time (PT) with international normalized ratio (INR) and serum fibrinogen level, which are recognized for their ability to predict VICC at an early stage and with better sensitivity (Sn) and specificity (Sp), in most rural settings where snakebite is treated, can lead to delays in diagnosis [ 6, 7]. 

The 20WBCT recommended by the World Health Organization (WHO) is the most routinely employed bedside screening tool for VICC assessment in India [ 8, 9]. It is the tool of choice due to its cost-effectiveness in low and middle-income countries (LMIC) [ 4, 7, 10]. The 20WBCT has low Sn for detecting coagulopathy in snake envenomation compared to conventional coagulation tests like PT and Activated Partial Thromboplastin Time (aPTT) [ 7, 11]. MLW is another test that has been employed for decades to assess coagulation in many hospitals in South India. It is an inexpensive bedside test used as part of the standard treatment protocol for snakebite victims [ 12]. We undertook the present study to determine the diagnostic utility of the MLW method in comparison with 20WBCT, at Emergency Department (ED) admission, amongst snakebite victims in central Kerala, Southern India. 

## Methods

This was a prospective observational unblinded diagnostic test study done among snakebite patients who presented to the ED of Jubilee Mission Medical College and Research Institute in South India during the study period (one year) after obtaining independent approval from the institutional review board and institutional ethics committee (01/19/IEC/JMMC&RI). The study was registered under clinicaltrials.gov (NCT03890016).

### Participants

**Figure 1.**
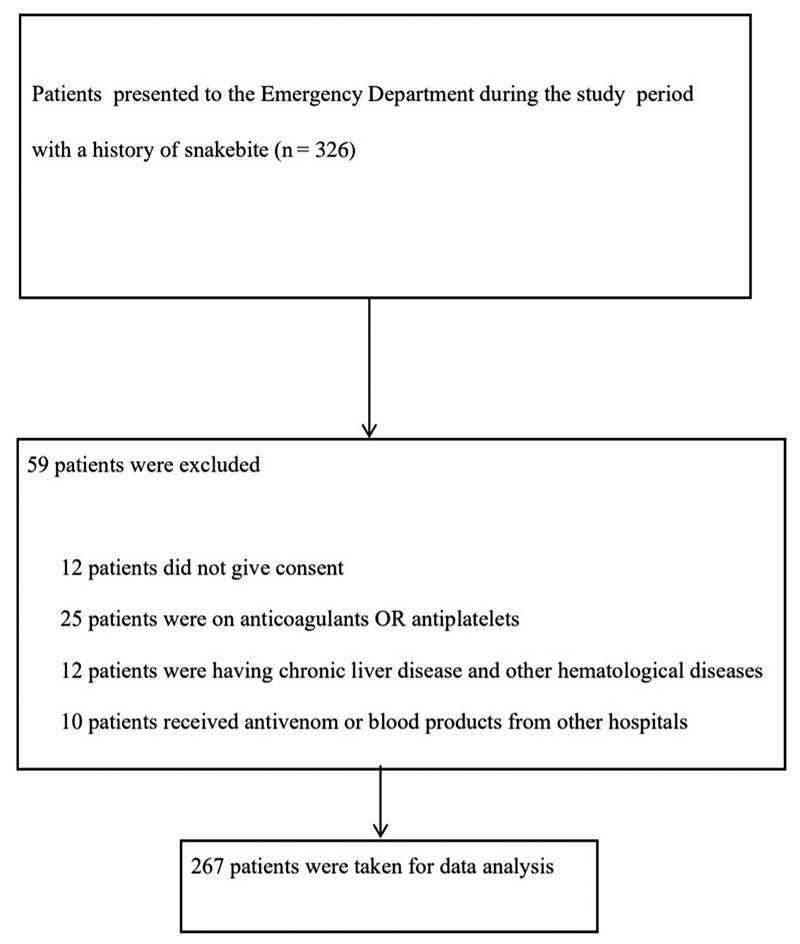
Flow chart depicting the selection of snake bite patients for the study after meeting the exclusion and inclusion criteria.

**Figure 2.**
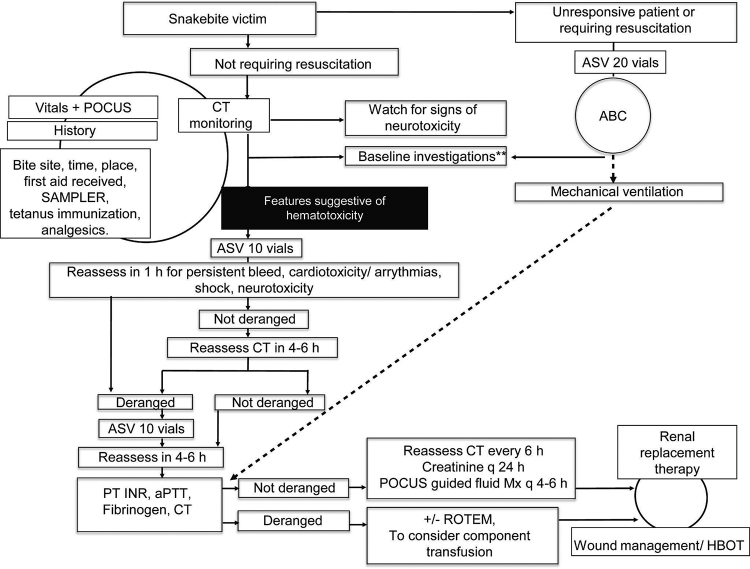
Institute (JMH ED) snakebite management protocol. aPTT: activated partial thromboplastin time; ABC: airway, breathing, and circulation; ASV: anti-snake venom; CT: clotting time (modified Lee and White test); HBOT: hyperbaric oxygen therapy; INR: international normalized ratio; JMH ED: Jubilee Mission Hospital Emergency Department; Mx: management; POCUS: point of care ultrasound; PT: prothrombin time; ROTEM: rotational thromboelastometry; SAMPLER: signs and symptoms, allergies, medications, past medical history, last meal, events leading to the present illness, and risk factors. **Baseline investigations include complete blood count, creatinine, PT INR, and aPTT. Additional blood tests are sent at admission at the discretion of the treating clinician on a case-by-case basis. JMH ED protocol reproduced with permission.

All patients with a ‘history of snakebite’ who presented to the ED during the study period were enrolled sequentially in the study after obtaining their consent. Patients with known bleeding disorders, those on anticoagulants/antiplatelets, individuals who received antivenom or blood products elsewhere, those with chronic liver disease/other hematologic disorders, and those who did not provide written informed consent were excluded from the study ( Figure 1). All snakebite patients presenting to the ED were managed as per the institute's snakebite treatment protocol ( Figure 2). 

History of snakebites, for the purpose of this study, was defined as ‘patients or their bystander claiming in a sound mind and not under the influence of any inebriants, to have witnessed being bitten by a snake’. 

### Procedure

Phlebotomists who received training made sure to collect blood samples in accordance with the institution's protocol. They performed collections at admission, one hour, three hours, and six hours after the bite, and then continued to collect samples every six hours up to 24 hours in all cases where envenomation was suspected.

The samples collected were assessed for coagulopathy by two methods at the bedside; 20WBCT and MLW. 20WBCT and MLW were done simultaneously by a trained phlebotomist. 20WBCT works on the principle that blood clots on exposure to glass, whereas MLW in addition employs agitation of the sample by tilting the tube every minute, which speeds up the clotting process. Samples for doing PT were also collected at the same time.

### 20WBCT

**Figure 3.**
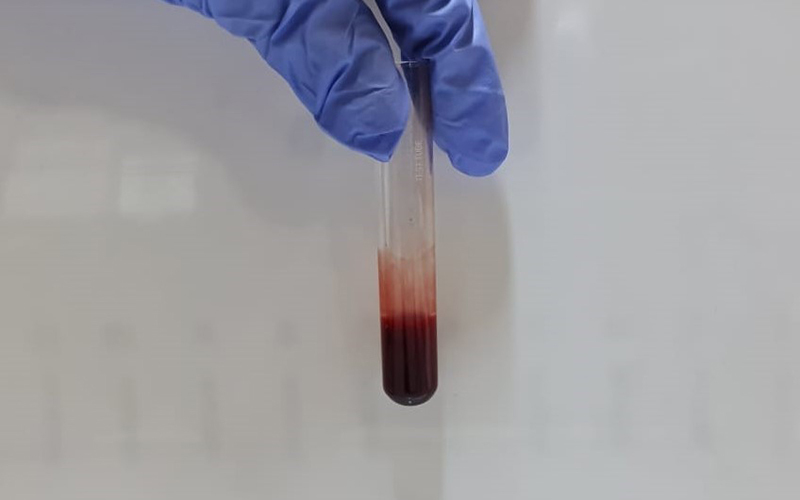
Blood sample for the Twenty-minute whole blood clotting test immediately after collection.

**Figure 4.**
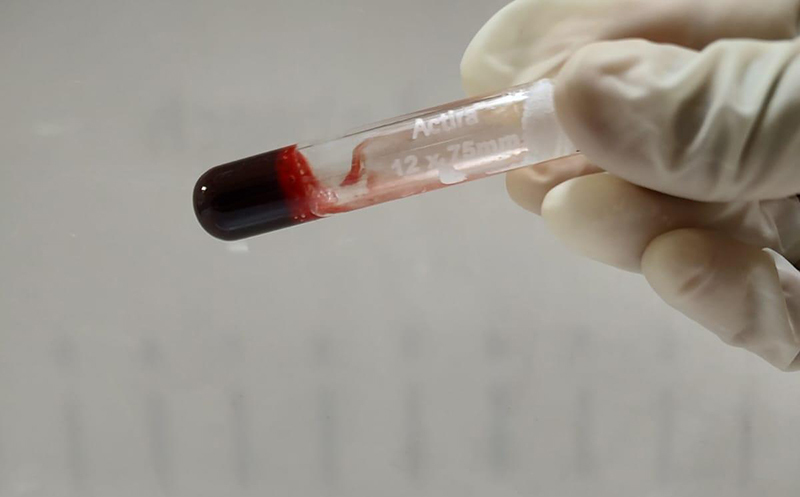
Twenty-minute whole blood clotting test. A solid clot is retained upon tilting of the tube at 20 minutes: normal result.

**Figure 5.**
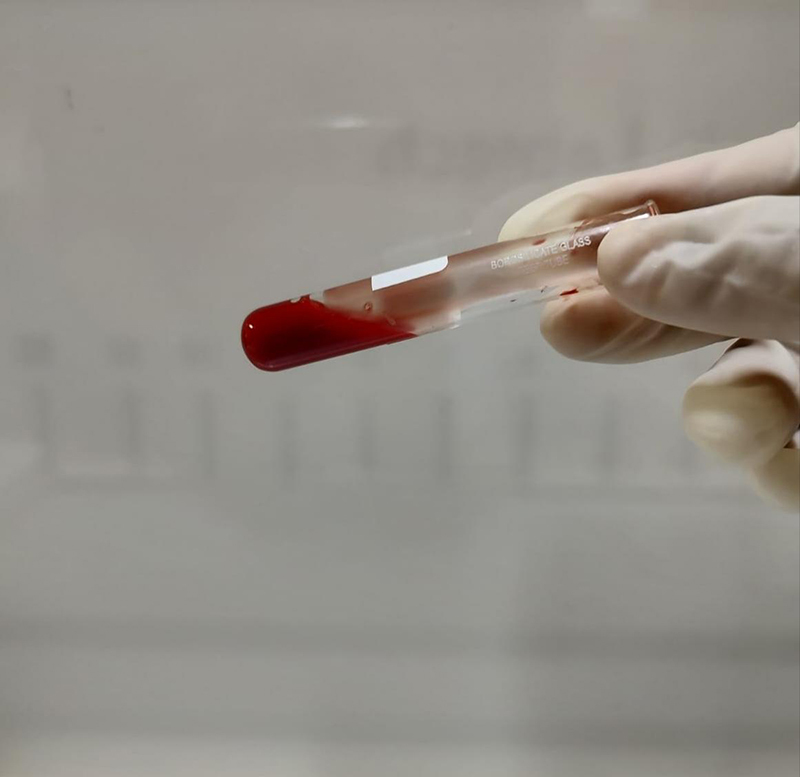
Twenty-minute whole blood clotting test. The blood is not clotted after 20 minutes: abnormal result.

To perform the 20WBCT, 2 ml of atraumatic venepuncture blood samples are collected in a new clean, dry, properly labeled, glass test tube (Borosilicate glass, 12*75mm) ( Figure 3). The blood sample is left undisturbed for a period of 20 minutes in this tube. At the end of 20 minutes, the sample is tilted approximately 60° to see if a stable clot has formed. A stable blood clot in a test tube for a 20WBCT test would appear as a solid mass at the bottom of the tube that maintains its shape and clings to the glass vessel without any breakage or dissolution on tilting the tube. The presence of a stable clot at the end of 20 minutes is noted as "normal" ( Figure 4) and if no stable clot is formed; it is noted as "abnormal" ( Figure 5). 

### MLW

**Figure 6.**
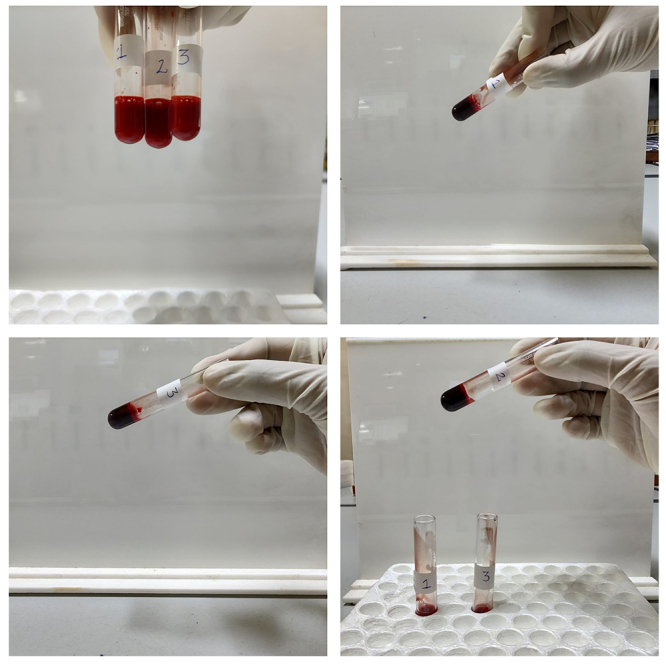
Modified Lee and White method. Samples in all three test tubes are clotted.

**Figure 7.**
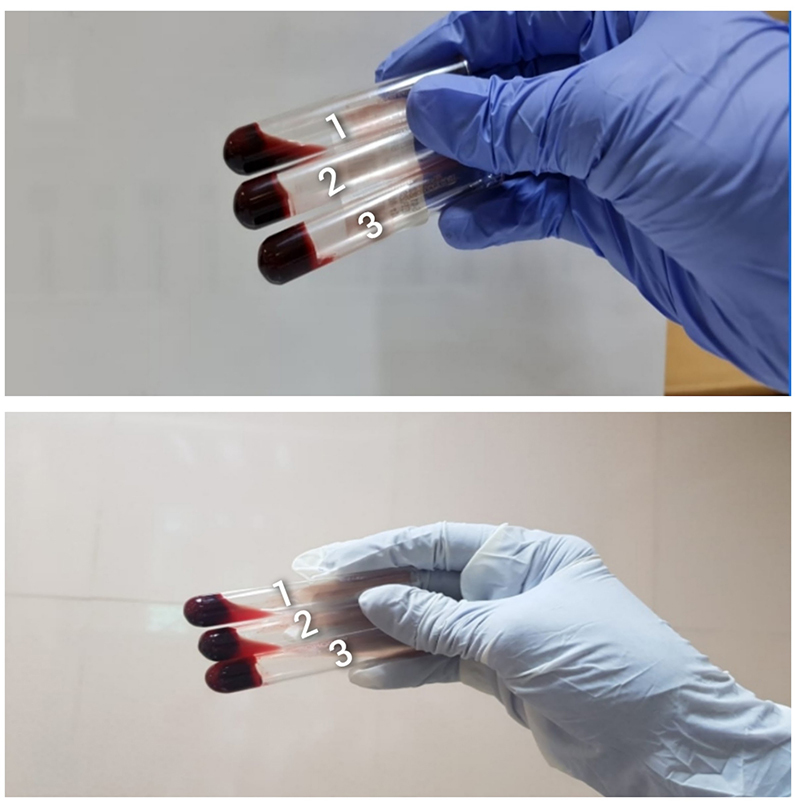
Modified Lee and White method. Samples showing clot lysis in test tube 1 (above) and test tubes 1 and 2 (below).

For MLW, 6 ml of atraumatic venepuncture blood sample is collected using a sterile plastic unlubricated syringe. Two ml each is dispensed into three new clean, dry, properly labeled, glass test tubes (Borosilicate, 12* 75mm), sequentially labeled T3, T2, and T1 respectively. The blood in T1 theoretically has the highest amount of Tissue Factor. The three test tubes are kept on a rack at ambient temperature. Although the emergency department and transfusion department where the study took place were air-conditioned, the temperature was not being monitored. Using a stopwatch; timing is started as soon as the last test tube (T1) is filled. The test tube (T1) is left undisturbed for five minutes, following which the tube T1 is tilted approximately 60° to check for clot formation. This is repeated every minute while the other tubes are left undisturbed. After the blood in the T1 test tube has clotted, the T2 would be tilted every minute and examined. Following its clotting, T3 would be examined and the timer would be stopped at the time when a clot is noted in T3. The total time is documented in minutes as the CT value ( Figure 6). Once clotted, the sample in T3 would be further assessed every minute for up to 10 minutes to look for breakup or lysis of the clot. Breakage or dissolution of a formed clot if seen was noted as “clot lysis” ( Figure 7). Absent clot formation at 20 minutes and clot lysis are both taken as prolonged MLW. 

### PT

Prothrombin time (PT) was done using an automated coagulation analyzer ACL-TOP 300, at admission for patients. Samples were collected in citrate tubes (Blue top, 3.2% sodium citrate) and centrifuged at the central lab. Platelet Poor Plasma was used for the test. Quality control was performed using the Pooled Normal Plasma daily as per the lab standards. Prolonged PT with an INR > 1.4 was taken as a surrogate standard for determining the presence of venom-induced consumption coagulopathy (VICC).

### Sample size and statistical analysis

Based on the data abstracted for a pilot study in the same institute by Melit et al the sample size was calculated as 267 using the confidence interval set at 95% with z value at 1.96, with a sensitivity of 82%, 50% prevalence and precision of 7% [ 13].

$$n=\frac{({Z_{a/2})}^{2\;*}sensitivity\left(1-sensitivity\right)}{d^{2*}prevalence}$$

We assessed the accuracy of the MLW and 20WBCT, with INR as the reference standard. Sensitivity (Sn), specificity (Sp), positive predictive value (PPV), negative predictive value (NPV), likelihood ratios (LR+, LR-), and accuracy were calculated with 95% confidence intervals (CI).

The data were analyzed using IBM SPSS statistics 64-bit MS Windows ver. 25.0.0.0 (IBM Corp., Armonk, N.Y., USA). Descriptive statistics were performed to examine the relation between MLW and 20WBCT with simultaneously drawn PT values at admission, as the surrogate gold standard.

## Results

**Figure 8.**
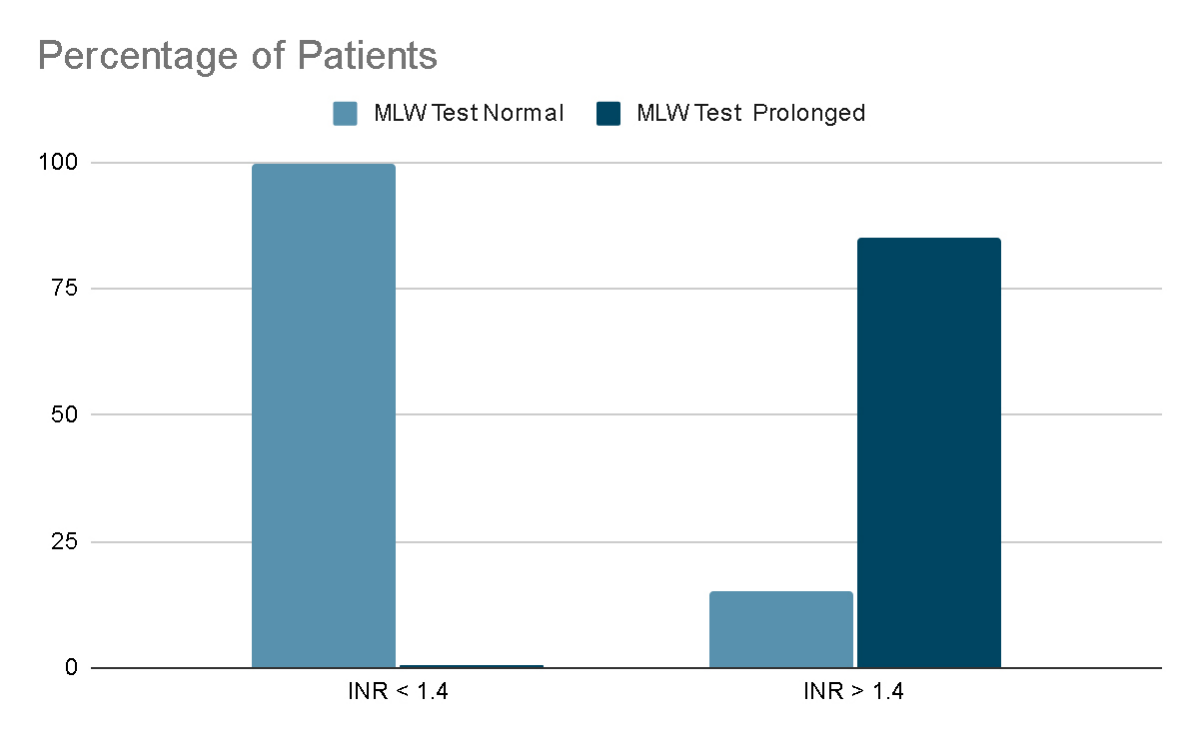
Proportion of modified Lee and White tests (%) that were positive or negative for INR values.

**Figure 9.**
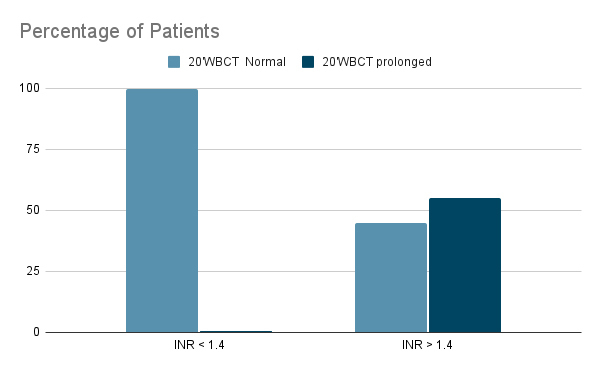
Proportion of twenty-minute whole blood clotting tests (%) that were positive or negative for INR values.

About 326 patients were presented to the ED during the study period out of which 267 patients met the inclusion and exclusion criteria. Out of 267 patients, 20 (7.5%) patients had VICC. Amongst those who had VICC (n = 20), MLW was prolonged for 17 (85.0%) patients ( Figure 8) whereas 20WBCT was abnormal for 11 (55.0%) patients ( Figure 9). While doing MLW, 2 patients showed clot lysis i.e., MLW samples clotted at 18 minutes but when observed further showed dissolution of the formed clot after 4 and 6 minutes respectively. 20WBCT was recorded as normal for these patients while simultaneously reported PT was prolonged (INR > 1.4). 

Out of 247 patients who had no coagulopathy detected at admission by CCT, MLW, and 20WBCT were falsely positive for the same 1 (0.4%) patient. MLW was 85% (95% CI: 61.1-96.0) sensitive and 99.6% (95% CI: 97.4-99.9) specific ( Table 1), whereas 20WBCT was 55% (95% CI: 32.04-76.17) sensitive and 99.6% (95% CI: 97.4-99.9) specific ( Table 2) in detecting coagulopathy amongst snakebite victims. 

MLW has a PPV of 94 (95% CI: 70.6-99.7) and an NPV of 98.8 (95% CI: 96.2-99.7%) and a positive likelihood ratio (LR+) of 85 and a negative likelihood ratio (LR-) of 0.15. It is 98.5% accurate ( Table 1). The 20WBCT has a PPV of 91.6 (95% CI: 59.8-99.6) and an NPV of 96.4 (95% CI: 93.2-98.3, LR+: 55, LR- of 0.45). The test has an accuracy of 96.2% in detecting VICC ( Table 2). Pre-defined post hoc analysis of MLW excluding clot lysis decreased its Sn to 83.3% (95% CI: 59-96) ( Tables 3 and 4). 

**Table 1.** Modified Lee and White (MLW) method compared with INR (INR > 1.4 = abnormal, CT > 20 = prolonged).

MLW test		INR > 1.4	INR < 1.4	Total
Prolonged	Count	17	1	18
	% within INR	85% (sensitivity) (95% CI: 61.1-96.0)	0.4%	6.7%
Normal	Count	3	246	249
	% within INR	15%	99.6% (specificity) (95% CI: 97.4-99.97)	93.3%
Total	Count	20	247	267
	% within INR	100.0%	100.0%	100.0%

Positive predictive value (PPV): 94 (95% CI: 70.6-99.7%); negative predictive value (NPV): 98.8 (95% CI: 96.2-99.7%); positive likelihood ratio (LR+): 85; negative likelihood ratio (LR-): 0.15; accuracy: 98.5%.

**Table 2.** Twenty-minute whole blood clotting test (20WBCT) compared with INR (INR > 1.4 = abnormal, 20WBCT > 20 = abnormal).

20WBCT		INR > 1.4	INR < 1.4	Total
Abnormal	Count	11	1	12
	% within INR	55% (sensitivity) (95% CI: 32.04-76.17)	0.4%	4.5%
Normal	Count	9	246	255
	% within INR	45%	99.6% (specificity) (95% CI: 97.4-99.9)	95.5%
Total	Count	20	247	267
	% within INR	100.0%	100.0%	100.0%

Positive predictive value (PPV): 91.6 (95%CI: 59.8-99.6); negative predictive value (NPV): 96.4 (95%CI: 93.2-98.3); positive likelihood ratio (LR+): 55; negative likelihood ratio (LR-): 0.45; accuracy: 96.2%.

**Table 3.** Modified Lee and White (MLW) method compared with INR after excluding clot lysis (INR > 1.4 = abnormal; CT > 20 = prolonged).

MLW test		INR > 1.4	INR < 1.4	Total
Prolonged	Count	15	1	16
	% within INR	83.3% (sensitivity) (95% CI: 59-96%)	0.4%	6.0%
Normal	Count	3	246	249
	% within INR	16.7%	99.6% (specificity) (95% CI: 97.7-99.9)	94.0
Total	Count	18	247	265
	% within INR	100.0%	100.0%	100.0%

**Table 4.** Twenty-minute whole blood clotting test (20WBCT) compared with INR after excluding clot lysis (INR > 1.4 = abnormal, 20WBCT > 20 = abnormal).

20WBCT		INR > 1.4	INR < 1.4	Total
Abnormal	Count	11	1	12
	% within INR	61.1% (sensitivity) (95% CI: 36-83)	0.4%	4.5%
Normal	Count	7	246	253
	% within INR	38.9%	99.6% (specificity) (95% CI: 97.7-99.9)	95.5
Total	Count	18	247	265
	% within INR	100.0%	100.0%	100.0%

## Discussion

In the absence of CCTs, bedside clotting tests represents a cost-effective and timely diagnostic test for clinicians to detect coagulopathy in snakebite victims, especially in a rural setting [ 4]. The 20WBCT introduced by Warrell et al. is a simple, relatively inexpensive, bedside test essentially a modification of the LWCT, routinely done in all snakebite patients in developing countries, especially in rural areas, where expensive tests like PT and APTT are often not available or affordable [ 9]. It is done to determine the presence of coagulopathy and also for the decision regarding the administration of ASV [ 8]. 

Although 20WBCT was intended for resource-limited settings, even hospitals capable of CCT (PT with INR, aPTT) use 20WBCT routinely to manage snake bite patients. The time to develop coagulopathy in individuals is dependent on multiple patient factors, the snake species, the amount of venom injected, and the time to the presentation at a hospital. This necessitates frequent sampling of the individual and the simplicity of taking a sample of blood in a glass tube, with no interventions, waiting for twenty minutes to check for a binary response as to which a clot is formed ‘yes’ or ‘no’, makes it a very cost-effective test. Although it is known to be specific, its sensitivity has been questioned in many studies. In an observational study from Sri Lanka of 140 cases of Russell’s viper bite patients, the 20WBCT showed a sensitivity of 40% and specificity of 100%. The study concluded that 20WBCT was not sensitive enough to detect severe degrees of coagulopathy and the administration of antivenom was delayed [ 4]. In a subsequent study, they included trained research assistants and used glass tubes with standard dimensions thereby standardizing 20WBCT which improved the sensitivity to 82% [ 4, 11]. The aggregate weighted sensitivity for 20WBCT at detecting prolonged PT at an INR > 1.4, from 12 studies (n = 2006) in a recent meta-analysis was 84% (95% CI 0.61 to 0.94) with a specificity of 91% (95% CI 0.76 to 0.97) [ 7]. Even though WBCT is the WHO - recommended bedside coagulation test to detect VICC, its low sensitivity and false negative results can lead to delayed administration of ASV in patients without clinical signs of envenomation. In our study, equally specific, MLW was more sensitive to detecting coagulopathy at admission in snakebite victims. 

Results in this study were comparable to a recent study conducted in Brazil, MLW was studied in Bothrops sp. envenomation which showed moderate sensitivity (78%) for MLW [ 14]. MLW gives a quantitative value for clotting ‘time’ whereas 20WBCT gives a binary response as to whether a clot is formed ‘yes’ or ‘no’. Although we initially hypothesized that serial MLW readings across multiple samples could result in a quantitative increase in time to clot as compared to 20WBCT aiding in early detection of coagulopathy, we were unable to demonstrate that, presumably because the study was underpowered for the same. 

Snake venom is a cocktail of proteinaceous components that results in various hematotoxic effects ranging from “prothrombotic” to the “hyperfibrinolytic” spectrum of coagulation. 20WBCT looks at the prothrombotic end of the spectrum whereas MLW looks at both [ 15, 16]. The MLW method continues to look for the stability of the clot and fibrinolysis in the form of clot lysis. MLW helps in the interpretation of coagulopathy from the same sample even if the 20WBCT shows a normal value at admission. Repeated/ delayed reading of 30 minutes proposed by Benjamin JM et al may circumvent this limitation in 20WBCT, however, this has not yet been replicated in other studies [ 17]. 

The evaluation and comparison of clotting time (MLW) with the clotting test (20WBCT) at different time frames, especially post-antivenom therapy, to detect normalization of coagulation and detecting secondary resumption of coagulopathy were beyond the scope of this study. Multiple tubes being used in MLW makes it more cumbersome for the novice, but eliminating the same, and employing the tilt and assessment for a single test tube and looking for clot lysis could combine the best of the two tests for a better bedside coagulation test, which may warrant further investigation.

### Limitations

The blood sample collection and the tests were performed by trained lab assistants (phlebotomists) at admission. Having a trained phlebotomist perform the procedure would inevitably result in a proficiency bias affecting the generalisability of the result. Routine errors in performing 20WBCT and MLW leading to false positivity in the results like improper timing, improperly cleaned tubes, cleaning solution remnants, detergent coated tubes, improper sample collection, and procedure, etc. are avoided and the test would be more standardized which could also explain the high specificity noted in the results. 

VICC was defined as prolonged PT (INR > 1.4) in this study and was not confirmed by other coagulation parameters like D-dimer and Fibrinogen levels. However previous studies support the use of INR values > 1.4 as a marker of VICC [ 1, 6, 7, 17]. The study, although stringently recruited consecutive cases of snakebite victims, was unable to recruit adequate snakebite victims with coagulopathy in the set time frame from a single center. 

The study follow-up of patients was limited to their time in ED (or up to 24 hours whichever was later) and there was a lack of details concerning responsible snake species. Mortality and morbidity outcome correlation were beyond the scope of our research question. 

The time taken for both MLW and 20WBCT is 20-30 mins. The major difference is the personnel involved. This was not quantified in terms of operational expenses. The study did not undertake the identification of snakes because of insufficient funding for specimen storage. Additionally, there was a valid concern that in the absence of antivenom or snake venom detection kits in the region, human-wildlife interaction could occur, leading to no change in patient treatment outcomes but potentially resulting in unnecessary snake killings. Nevertheless, it is the authors' firm belief that a comprehensive understanding of the local snakes is essential to comprehend the distinctions in the clinical manifestation of hematotoxic snakebites in the region. Future research should include species identification, larger sample sizes to account for venomous bites, and a cost-effectiveness analysis to assess the economic impact of the test.

## Conclusion

In a setting with standardized training, the MLW test is more sensitive in detecting VICC when compared to 20WBCT amongst snakebite patients. However, to consolidate these findings, further studies involving larger sample sizes, multiple snakebite treatment centers, and a substantial number of envenomed patients are necessary. Additionally, extending the interpretation time of the 20WBCT through continued reading of the blood sample, along with exploring comprehensive analysis of additional hematological parameters, holds potential for improving the study's robustness. Our ongoing efforts aim to validate these results and provide a more comprehensive understanding of this subject [ 18]. 

## Abbreviations

20WBCT: 20-minute Whole Blood Clotting Test; MLW: Modified Lee and White; VICC: Venom-induced consumption coagulopathy; VCT: Venous clotting Time; PT: Prothrombin Time; INR: International Normalized Ratio; aPTT: Activated Partial Thromboplastin time; WHO: World Health Organization; LMIC: low and middle-income countries.
